# Hypoxic burden reduction after lateral pharyngoplasty in patients with obstructive sleep apnea: Beyond the apnea-hypopnea index

**DOI:** 10.1007/s11325-026-03808-7

**Published:** 2026-09-22

**Authors:** André Luís Pereira Vieira, Bruno Bernardo Duarte, Aurelio Rochael Almeida, Mário Edvin Greters, Vânia Aparecida Leandro-Merhi, José Luís Braga de Aquino

**Affiliations:** 1 Pontifícia Universidade Católica de Campinas (PUC-Campinas), Campinas, São Paulo, Brazil; 2 Universidade Estadual de Campinas (UNICAMP), Campinas, São Paulo, Brazil

**Keywords:** Obstructive sleep apnea, Hypoxic burden, Lateral pharyngoplasty, Apnea-hypopnea index, Polysomnography

## Abstract

**Purpose:**

The apnea-hypopnea index (AHI), traditionally used to assess obstructive sleep apnea (OSA) severity and surgical outcomes, has limitations in reflecting cardiovascular risk and pathophysiological burden. Although lateral pharyngoplasty has demonstrated favorable outcomes regarding AHI reduction, its impact on hypoxic burden (HB) remains unknown. This study evaluated HB changes after lateral pharyngoplasty in patients with OSA, including those classified as surgical failures according to Sher’s criteria.

**Methods:**

This observational, longitudinal study with a mixed retrospective and prospective design included 27 adults with OSA who underwent lateral pharyngoplasty (version 6) between 2016 and 2025, with complete preoperative and postoperative polysomnographic data for HB assessment. HB was calculated using validated commercial software. Nonparametric tests were used for intra- and intergroup comparisons. Surgical success was defined according to Sher’s criteria.

**Results:**

A significant reduction in HB was observed across all variants: total sleep time (median from 97.1%·min/h to 35.0%·min/h, *p* < 0.0001), REM sleep, NREM sleep, and supine and non-supine positions, with a significant reduction in median AHI (median from 33.8 to 12.3 events/h, *p* < 0.0001). Even among surgical failures according to Sher’s criteria, HB decreased significantly (median from 102.5%·min/h to 67.3%·min/h, *p* = 0.0107), suggesting pathophysiological improvement not captured by AHI alone.

**Conclusion:**

HB is a potentially more sensitive metric to assess therapeutic response in OSA, incorporating multiple dimensions of nocturnal hypoxemia beyond AHI. Lateral pharyngoplasty promoted consistent HB reduction, including in surgical failures, supporting HB as a complementary tool for therapeutic evaluation, with potential applications in cardiovascular risk stratification.

## Introduction

Obstructive sleep apnea (OSA) is a disease characterized by intermittent collapse of the upper airway (UA), reduced airflow, sleep fragmentation, intermittent blood gas disturbances (hypoxemia and hypercapnia), and recurrent awakenings during sleep [1]. Epidemiological studies have demonstrated a high prevalence of OSA in the adult population, ranging from 38.2% to 83.8%, considering an apnea-hypopnea index (AHI) > 5 events/h [2, 3]. The pathophysiology of OSA is multifactorial, and airway collapse occurs predominantly in the lateral pharyngeal wall [4].

The clinical presentation of OSA includes nocturnal and daytime symptoms associated with reduced quality of life and increased cardiovascular risk due to chronic exposure to a cascade of physiological changes. The diagnosis and severity classification of the disease are traditionally based on AHI, an index that quantifies only the frequency of respiratory events. However, evidence suggests that symptom severity, as well as cardiovascular outcomes and mortality, are not directly proportional to AHI. Furthermore, historical changes in the definition of respiratory events hamper comparisons between studies conducted in different periods [5].

The ability of AHI to diagnose OSA and classify its severity has increasingly been questioned, as evidence suggests that cardiovascular risk is more closely related to the intensity and duration of desaturation/reoxygenation events than to the frequency of respiratory events per se [6, 7].

Thus, the concept of hypoxic burden (HB) was proposed, defined as the area under the oxygen desaturation curve associated with obstructive respiratory events, capturing not only event frequency but also event duration and intensity [7, 8]. Recent studies have demonstrated a consistent association between elevated HB and increased cardiovascular risk, heart failure, and mortality [9]. Unlike HB, elevated ODI was not associated with severe cardiovascular outcomes [10]. Labarca et al. [11] observed that each one-standard-deviation increase in HB was associated with increased risk of severe cardiovascular disease, cardiovascular events, all-cause mortality, and cerebrovascular disease.

Azarbarzin et al. [12], evaluating the osteoporotic fractures in men study (MrOS) and Sleep Heart Health Study (SHHS) cohorts, demonstrated an association between elevated HB and increased cardiovascular and all-cause mortality when HB ≥ 53%·min/h. In the SHHS cohort, increased cardiovascular mortality risk was observed when HB ≥ 88%·min/h. In contrast, AHI was not associated with cardiovascular mortality in either cohort.

In a post hoc analysis of the International Study of Comparative Health Effectiveness with Medical and Invasive Approaches (ISAACC) study [13], patients with HB above 73%·min/h who adhered to continuous positive airway pressure (CPAP) treatment experienced a reduction in cardiovascular complications. This association was not observed when considering traditional metrics such as AHI, ODI, or T90 [8].

The gold-standard treatment for OSA remains positive airway pressure (PAP) therapy; however, poor treatment adherence has led to the development of therapeutic alternatives. Uvulopalatopharyngoplasty (UPPP) was one of the first widely disseminated surgical techniques [14], but demonstrated better outcomes only in selected patients, particularly those with tonsillar hypertrophy and low modified Mallampati scores, who represent approximately 10% of patients with OSA [15].

To address both anatomical and pathophysiological aspects of OSA, lateral pharyngoplasty (LP) was developed [16]. The procedure consists of reconstructing the lateral pharyngeal wall through myotomy of the superior pharyngeal constrictor and palatopharyngeus muscles, followed by suturing of laterally pedicled flaps to the palatoglossus muscle. This technique significantly reduces constrictor tension in the lateral pharyngeal walls, allowing laterolateral and anteroposterior enlargement, in addition to increasing caudal traction of the pharynx through flap repositioning [17].

Recent studies have demonstrated surgical success rates close to 70% with LP, as well as improvements in minimum and mean oxyhemoglobin saturation [18]. However, these studies used Sher’s criteria, proposed in 1996, as the parameter for surgical success, defining success as a reduction greater than 50% in preoperative AHI combined with postoperative AHI < 20 events/h [19].

To date, no studies have evaluated HB behavior in patients undergoing LP, nor which metric would be most appropriate to define surgical success regarding cardiovascular risk reduction. Therefore, the objectives of this study were to evaluate HB behavior in patients undergoing LP for OSA treatment and to compare HB behavior between surgical success and surgical failure groups according to Sher’s criteria.

## Methods

This was an observational, analytical, longitudinal study with a mixed retrospective and prospective design conducted in adult patients with OSA who underwent lateral pharyngoplasty. The study was approved by the Research Ethics Committee of Pontifícia Universidade Católica de Campinas, São Paulo, Brazil, under approval number 7.126.817 (CAAE: 83167724700005481), and was conducted at the Department of Otorhinolaryngology of Hospital PUC-Campinas.

The study population consisted of patients aged ≥ 18 years diagnosed with OSA, defined by an AHI ≥ 5 events/h, who underwent lateral pharyngoplasty version 6 between 2016 and 2025, performed under the supervision of the same otorhinolaryngologist specializing in sleep medicine.

Surgical indication was assigned to patients with poor adherence to positive airway pressure (PAP) therapy, body mass index (BMI) < 35 kg/m², preserved palatine tonsils, and no clinical contraindication or increased surgical risk.

Inclusion criteria were symptoms of obstructive sleep-disordered breathing, poor adherence or refusal to use PAP therapy, and preoperative and postoperative type I polysomnography performed with a minimum follow-up interval of six months. Patients with previous oropharyngeal surgery, polysomnographic recordings incompatible with HB calculation, loss to follow-up, use of muscle relaxant medications, or untreated hypothyroidism were excluded.

### Study selection process

**Fig. 1:**
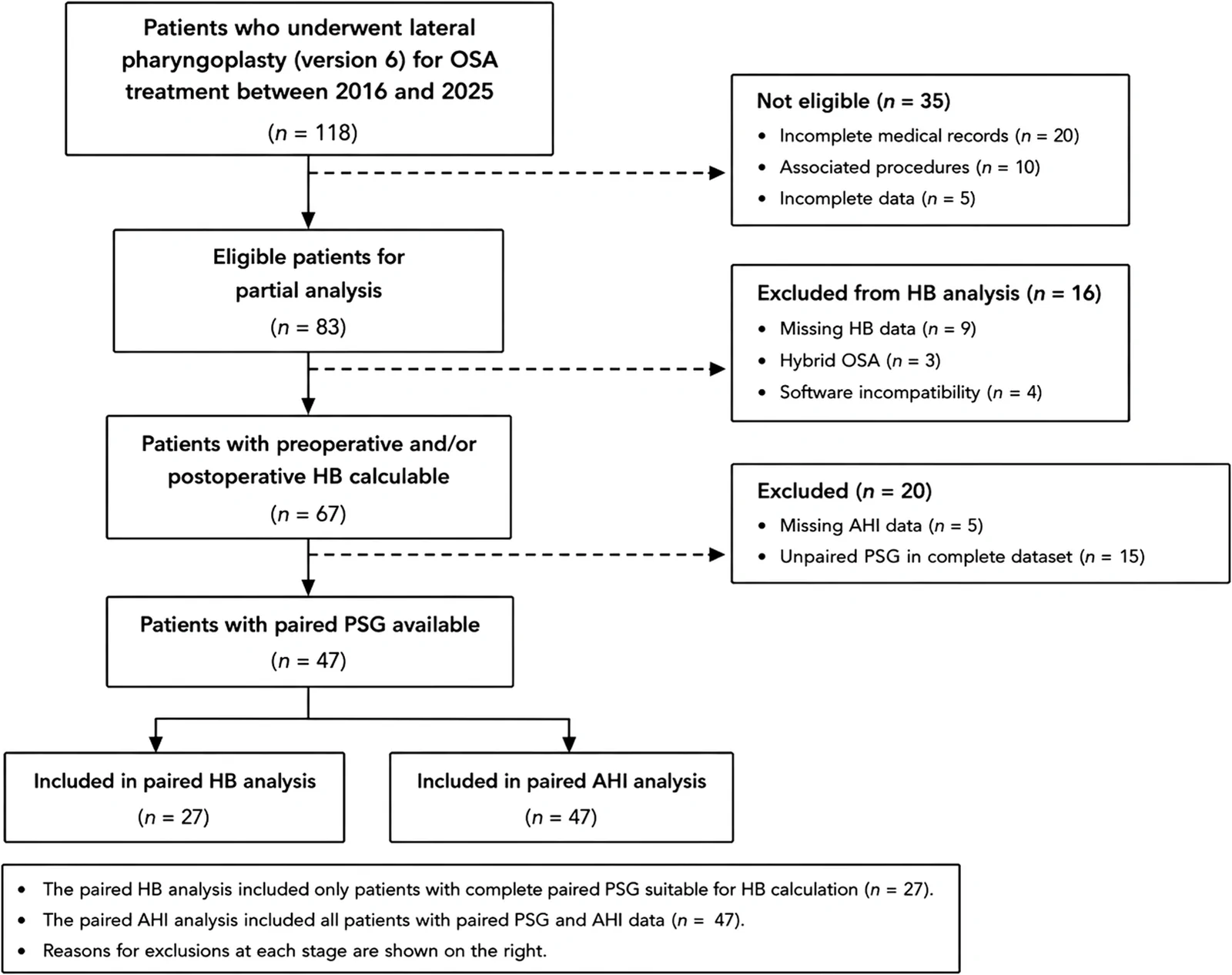
Sample selection

Patient selection was performed sequentially according to eligibility criteria and the availability of polysomnographic data. Paired AHI analysis included patients with available preoperative and postoperative PSG recordings, whereas paired HB analysis was restricted to those with complete paired recordings suitable for HB calculation. The sequential selection process is presented in Fig. 1.

### Polysomnographic assessment and hypoxic burden calculation

All patients underwent type I polysomnography at the same sleep laboratory, with examinations interpreted by the same pulmonologist specializing in sleep medicine, thereby reducing variability. Examinations were performed preoperatively and at least six months after surgery.

HB was calculated using SleepWare G3 software (Respironics, Philips), validated for HB calculation, using the area under the oxygen desaturation curve associated with previously identified obstructive respiratory events. Examinations performed before the routine implementation of this metric were reprocessed using a compatible software version.

Patients were classified into surgical success and surgical failure groups according to Sher’s criteria, defined as a ≥ 50% reduction in preoperative AHI combined with postoperative AHI < 20 events/h.

### Surgical technique

The surgical technique used in this study was LP version 6, consisting of the following steps. After tonsillectomy with preservation of the palatopharyngeus muscle, a myomucosal triangle was removed from the supratonsillar region to increase exposure of the tonsillar fossa. Myotomy of the superior pharyngeal constrictor muscle was performed in its cranial portion. The palatopharyngeus muscle was separated from the superior pharyngeal constrictor muscle, creating a myomucosal flap. The flap was repositioned using three Donatti (vertical mattress) sutures between the palatopharyngeus and palatoglossus muscles, encompassing the pharyngeal constrictor muscle. Myotomy of the caudal portion of the palatopharyngeus muscle was then performed. The flap sutures were tied, and a vertical release incision was made between the lateral and posterior walls of the oropharynx adjacent to the palatopharyngeus flap. All steps were repeated on the contralateral side [20].

### Statistical analysis

Categorical variables were presented as absolute frequencies and percentages. Numerical variables were described as mean ± standard deviation and median (minimum–maximum). The Mann–Whitney test was used for comparisons between independent groups, while preoperative and postoperative comparisons were performed using the Wilcoxon signed-rank test for paired samples. Effect sizes for paired comparisons were expressed as rank-biserial correlations. Where appropriate, 95% confidence intervals were calculated. Proportions were compared using Fisher’s exact test. Statistical significance was set at *p* < 0.05.

## Results

After chart review, 118 patients who underwent lateral pharyngoplasty version 6 for OSA treatment between 2016 and 2025 were identified. Following sequential eligibility and data availability assessment, 83 patients were eligible for partial analysis. Of these, HB could be calculated from at least one preoperative or postoperative PSG recording in 67 patients. Paired preoperative and postoperative PSG recordings were available for longitudinal analysis in 47 patients, all of whom were included in the paired AHI analysis. Among these patients, paired preoperative and postoperative HB values were available for 27 patients, who were included in the paired HB analysis. Thus, the paired HB cohort represented a subset of the paired AHI cohort. The complete selection process and reasons for exclusion are presented in Fig. 1.

 The descriptive characteristics of the sample is presented in Table 1. There was a predominance of male patients (82.2%), median age of 36 years, and mean preoperative body mass index of 29.15 ± 3.21 kg/m². The median preoperative AHI was 33.8 events/h, consistent with severe OSA.

**Table 1:** Descriptive characterization of the study sample: clinical, anthropometric, and polysomnographic respiratory characteristics

Variable	Preoperative		
	Mean ± SD or %	N	Median (min–max)
Age (years)	35.93±9.78	29	36.00 (13.00–54.00)
BMI (kg/m²)	29.15 ± 3.21	16	29.13 (23.55–34.90)
Brodsky tonsil grade I/II	67.44%	43	
Brodsky tonsil grade III/IV	32.56%	43	
Friedman I	31.0%	42	
Friedman II	31.0%	42	
Friedman III	38.0%	42	
T90 (%)	10.9 ± 19.9	38	5.0 (0.00–84.00)
AHI	39.7 ± 25.3	47	33.8 (5.2–114.5)
RDI	47.52±29.68	43	42.20 (6.10–105.00)
HB-TST	161.82±150.31	43	107.0 (9.40–659.20)
HB-REM	211.79±246.53	42	135.70 (0.00–1117.80)
HB-NREM	144.84±127.03	43	97.20 (9.70–449.40)
Supine HB	213.68±168.19	42	168.60 (0.00–747.20)
Non-supine HB	117.39±153.26	42	40.05 (0.00–667.70)

*HB-NREM* Hypoxic burden in NREM sleep, *HB-REM* Hypoxic burden in REM sleep, *HB TST* Hypoxic burden in total sleep time, *HB* Hypoxic burden, *brodsky tonsil grade* Palatine tonsil grade according to the Brodsky classification, *AHI* Apnea-Hypopnea Index, *RDI *Respiratory Disturbance Index, *BMI* Body Mass Index, *T90* Percentage of total sleep time with oxygen saturation <90%

**Fig. 2:**
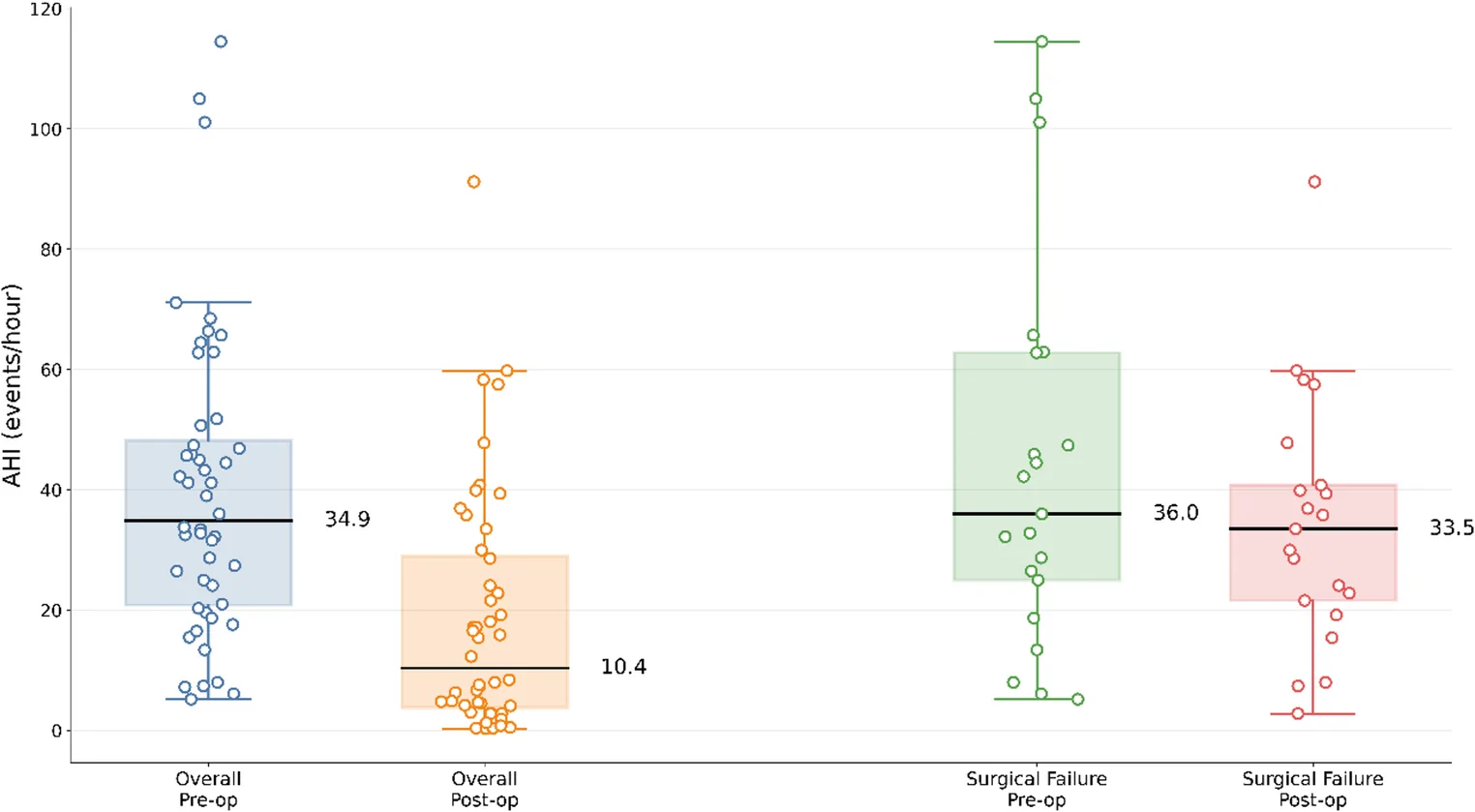
Boxplot with scatter points: AHI reduction in the overall group and in the failure group according to the Sher criteria

 In the preoperative and postoperative analysis, a statistically significant reduction in AHI and respiratory disturbance index (RDI) was observed after LP, with median AHI decreasing from 33.8 to 12.3 events/h (*p* < 0.0001) and median RDI decreasing from 33.6 to 15.6 events/h (*p* < 0.0001) (Table 2). The distribution of individual preoperative and postoperative AHI values in the overall group and in the surgical failure subgroup is shown in Fig. 2. According to Sher’s criteria, 55.3% of patients achieved surgical success.

**Table 2:** Comparison of polysomnographic respiratory variables between preoperative and postoperative periods

Variable	Preoperative		Postoperative		Δ		95% CI of Δ	Effect size	*p*-value (Δ)
	Mean ± SD	Median (min–max)	Mean ± SD	Median (min–max)	Mean ± SD	Median (min–max)			
T90 (*N* = 38)	10.9 ± 19.9	**5.0 (0–84.0)**	4.9 ± 11.1	**0.20 (0–43.50)**	−5.9 ± 21.1	**−1.5 (−84.0–37.1)**	−12.9 to 1.0	−0.58	**0.0018**
AHI (*N* = 47)	39.7 ± 25.3	**33.8 (5.2–114.5)**	18.9 ± 20.1	**12.3 (0.3–91.2)**	−20.8 ± 22.5	**−17.2(−74.6−23.3)**	−27.4 to −14.2	−0.91	**< 0.0001**
RDI (*N* = 27)	40.5 ± 25.3	**33.6 (6.1–105.00)**	21.6 ± 22.59	**15.6(0.5–91.2)**	−18.9 ± 20.1	**−12.8 (−64.4–11.90)**	−26.8 to −10.9	−0.87	**< 0.0001**
HB-TST (*N* = 27)	125.1 ± 117.9	**97.1(9.4–482.5)**	55.9 ± 77.3	**35.00 (0.40–359.20)**	−69.3 ± 84.7	**−53.1 (−424.0–24.6)**	−102.8 to −35.8	−0.92	**< 0.0001**
HB-REM (*N* = 26)	173.9 ± 216.2	**107.9 (0–901.5)**	46.5 ± 82.9	**16.50 (0–400.00)**	−127.5 ± 183.3	**−70.2 (−848.2−44.2)**	−201.5 to −53.5	−0.89	**< 0.0001**
HB-NREM (*N* = 27)	111.5 ± 100.0	**85.1 (9.7–449.4)**	56.5 ± 81.2	**32.3 (0–353.40)**	−55.0 ± 55.8	**−47.1 (−236.3–20.90)**	−77.0 to −32.9	−0.93	**< 0.0001**
Supine HB (*N* = 26)	170.6 ± 121.6	**158.2 (0–553.70)**	84.6 ± 113.20	**44.4 (0–481.80)**	−86.0 ± 95.7	**−91.1 (−317.3–121.40)**	−124.7 to −47.3	−0.76	**< 0.0001**
Non-supine HB (*N* = 27)	99.4 ± 129.8	**39.4 (0.30–667.7)**	37.2 ± 72.5	**12.1 (0–352.80)**	−62.3 ± 129.8	**−15.5 (−634.0–22.50)**	−113.6 to −10.9	−0.77	**< 0.0001**

*HB* Hypoxic burden, *HB-NREM* Hypoxic burden during NREM sleep. Comparison performed using the Wilcoxon test for paired samples; *HB-REM* Hypoxic burden during REM sleep, *HB TST* Hypoxic burden during total sleep time, *AHI* Apnea-Hypopnea Index, *RDI* Respiratory Disturbance Index, *T90* Percentage of total sleep time with oxyhemoglobin saturation below 90%, *95% CI* 95% confidence interval of the mean difference (Δ). Effect size: rank-biserial correlation. All HB values are expressed as %·min/h.

¹Non-parametric distribution; ²p-value: statistical significance p<0.05. Δ: difference between postoperative and preoperative values (post−pre)

**Fig. 3:**
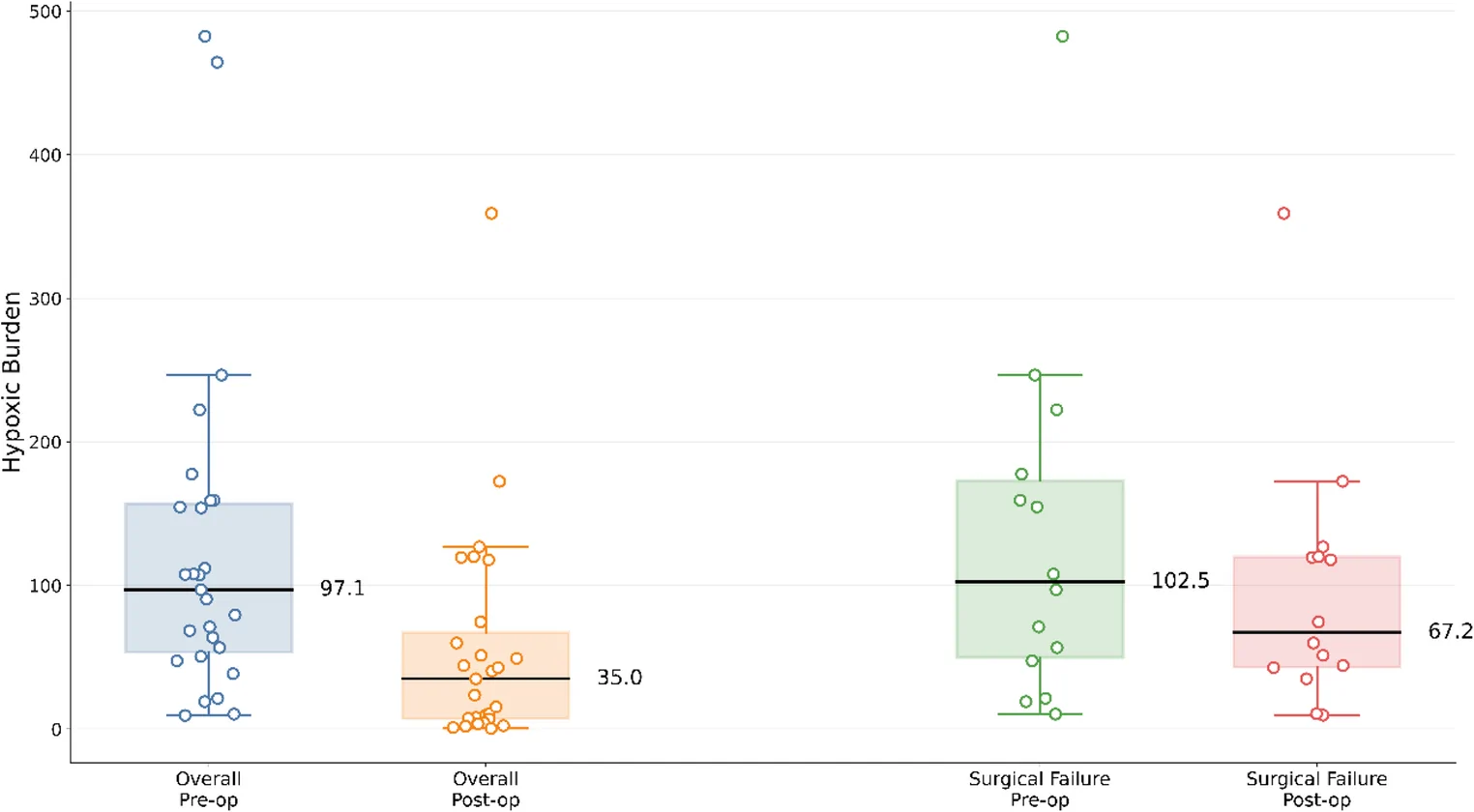
Boxplot with scatter points: HB TST reduction in the overall group and in the failure group according to the Sher criteria

 HB was analyzed during total sleep time (HB-TST), REM sleep (HB-REM), NREM sleep (HB-NREM), supine position, and non-supine position. A statistically significant reduction was observed in all analyzed variables, with median HB-TST decreasing from 97.1%·min/h to 35.0%·min/h (*p* < 0.0001), HB-REM from 107.9%·min/h to 16.5%·min/h (*p* < 0.0001), HB-NREM from 85.1%·min/h to 32.3%·min/h (*p* < 0.0001), supine HB from 158.2%·min/h to 44.4%·min/h (*p* < 0.0001), and non-supine HB from 39.4%·min/h to 12.1%·min/h (*p* < 0.0001) (Table 2). The distribution of individual preoperative and postoperative HB-TST values in the overall group and in the surgical failure subgroup is shown in Fig. 3.

 Table 3 presents the comparison between clinical, anthropometric, and polysomnographic variables according to surgical success and surgical failure based on Sher’s criteria. Despite classification as surgical failure according to Sher’s criteria, significant reductions were observed in HB-TST, HB-REM, HB-NREM, and non-supine HB.

**Table 3:** Comparison of clinical, anthropometric, and polysomnographic variables between patients with surgical success and surgical failure according to Sher’s criteria

Variable	Surgical failure	Surgical success	*p-value*
	Mean ± SD/Median (min–max)	Mean ± SD/Median (min–max)	
Brodsky tonsil grade I/II Brodsky tonsil grade III/IV	15 (75.0%) 5 (25.0%)	15 (60.9%) 9 (39.1%)	0.1184^**2**^
Preoperative T90	9.33 ± 15.34 (N=17)/4.90 (0.00–52.80)	12.42 ± 23.83 (N=20)/4.55 (0.00–84.00)	0.9878¹
Δ T90	1.61 ± 15.43 (N=17)/−0.10 (−38.50 - 37.10)	−12.32 ± 23.85 (N=20)/−4.35 (−84.00 - 0.10)	0.0537
Preoperative AHI	44.03 ± 31.78 (N=21)/36.00 (5.20–114.50)	36.23 ± 18.61 (N=26)/33.60 (7.20–71.10)	0.7081¹
Δ AHI	−9.70 ± 23.03 (N=21)/−6.40 (−74.60 - 25.30)	−29.77 ± 17.82 (N=26)/−27.95 (−64.40 −6.60)	**0.0002¹**
Preoperative RDI	45.15 ± 30.36 (N=14)/41.15 (6.10–105.00)	35.51 ± 18.45 (N=13)/33.40 (9.70–68.50)	0.6448¹
Δ RDI	−9.32 ± 17.40 (N=14)/−6.65 (−56.40 - 11.90)	−29.19 ± 18.07 (N=13)/−27.00 (−64.40 to- 9.20)	**0.0017**
Preoperative N3 (%)	13.33 ± 11.73 (N=14)/11.25 (0.00–43.90)	15.62 ± 6.94 (N=13)/16.30 (6.80–30.30)	0.3439¹
Δ N3 (%)	0.26 ± 11.83 (N=14)/2.65 (−27.60 - 22.10)	5.02 ± 6.72 (N=13)/1.70 (−3.70 - 14.40)	0.3198¹
Preoperative HB-TST	133.86 ± 125.74 (N=14)/102.50 (10.40–482.50)	115.76 ± 113.09 (N=13)/90.40 (9.40–464.40)	0.6448¹
Δ HB-TST	−37.87 ± 45.74 (N=14)/−24.00 (−123.30 - 24.60)	−103.08 ± 104.28 (N=13)/−72.00 (−424.00 −9.00)	**0.0240¹**
Percentage reduction in HB	10.53 ± 73.84 (N=14)/25.27 (−236.54 −57.97)	89.11 ± 11.43 (N=13)/91.30 (56.16–99.35)	**<0.0001**
Preoperative HB-REM	174.86 ± 198.34 (N=14)/109.05 (3.00–715.20)	172.88 ± 244.36 (N=12)/104.45 (0.00–901.50)	0.8170¹
Δ HB-REM	−102.90 ± 130.34 (N=14)/−62.65 (−404.90 - 44.20)	−156.18 ± 233.70 (N=12)/−77.75 (−848.20 - 2.10)	0.7381¹
Preoperative HB-NREM	127.55 ± 124.03 (N=14)/95.75 (9.70–449.40)	94.13 ± 66.26 (N=13)/81.60 (11.80–268.90)	0.7524¹
Δ HB-NREM	−28.67 ± 37.66 (N=14)/−13.35 (−96.90- 20.90)	−83.30 ± 59.36 (N=13)/−76.40 (−236.30 −11.80)	**0.0071¹**
Preoperative Supine HB	187.28 ± 143.22 (N=13)/157.40 (19.30–553.70)	153.83 ± 98.55 (N=13)/159.00 (0.00–386.30)	0.7976¹
Δ Supine HB	−37.33 ± 77.11 (N=13)/−71.90 (−135.90 - 121.40	−134.67 ± 89.44 (N=13)/−154.50 (−317.30 −6.40)	**0.0077¹**
Preoperative Non-supine HB	112.48 ± 121.78 (N=14)/73.40 (4.50–424.30)	85.41 ± 180.36 (N=13)/27.10 (0.30–667.70)	0.1204¹
Δ Non-supine HB	−49.63 ± 74.82 (N=14)/−8.90 (−246.60 to −0.90)	−75.85 ± 173.41 (N=13)/−25.70 (−634.00 −22.50)	1.0000¹
Preoperative BMI	30.76 ± 3.36 (N=8)/31.78 (25.20–34.90)	27.55 ± 2.67 (N=8)/26.99 (23.55–30.90)	** 0.0499¹**
Δ BMI	0.02 ± 0.91 (N=8)/−0.06 (−1.06 – 1.67)	−0.49 ± 2.00 (N=7)/−0.75 (−2.96 – 3.29)	0.2810**¹**

*HB * hypoxic burden, *NREM HB * hypoxic burden during non-rapid eye movement sleep. Comparison performed using the Wilcoxon signed-rank test for paired samples; *REM HB * hypoxic burden during rapid eye movement sleep, *TST HB * hypoxic burden during total sleep time, *PTG* Palatine Tonsil Grade, *AHI* Apnea–Hypopnea Index, *RDI* Respiratory Disturbance Index, *T90* Percentage of total sleep time with oxyhemoglobin saturation below 90%

¹Comparison performed using the Mann–Whitney test. ²Chi-square test. Δ: difference between postoperative and preoperative values (post − pre). Bold values indicate statistical significance (p < 0.05)

In the evaluation of pharyngeal anatomy, no statistically significant association was observed between palatine tonsil grade and surgical success (*p* = 0.1184). Both patients with non-hypertrophic and hypertrophic tonsils demonstrated postoperative reductions in AHI and HB.

When analyzed separately, the surgical failure group, despite not achieving sufficient AHI reduction to meet Sher’s criteria, demonstrated significant reductions in HB-TST, HB-REM, and HB-NREM. HB-TST showed a median reduction from 102.5 preoperatively to 67.3 postoperatively (*p* = 0.0107), while HB-REM decreased from 109.1 to 29.5%·min/h (*p* = 0.0085) and HB-NREM from 95.8 to 70.6%·min/h (*p* = 0.0085). Regarding body position, a significant reduction in non-supine HB was observed, with the median decreasing from 73.4 to 14.1%·min/h (*p* = 0.0001), whereas supine HB did not demonstrate a statistically significant reduction (*p* = 0.1677) (Table 4).

**Table 4:** Comparison of preoperative and postoperative hypoxic burden in the subgroup with surgical failure according to Sher´s criteria (*N* = 14)

Variable	Preoperative			Postoperative/		Δ			95% CI of Δ		Effect size		*p*-value^2^ (Δ)
	Mean ± SD		Median ^1^ (min–max)	Mean ± SD	Median^1^ (min–max)	Mean ± SD	Median^1^ (min–max)						
AHI		44.0 ± 31.8	**36.0 (5.2–114.5)**	34.3 ± 20.9	**33.5 (2.8–91.2)**	−9.7 ± 23.0	**−6.4 (− 74.6–25.3)**	−20.2 to 0.8		−0.43		0.0888	
HB-TST		133.9 ± 125.7	**102.5 (10.4–482.50)**	96.0 ± 90.1	**67.3 (9.60–359.20)**	−37.9 ± 45.7	**−24.0 (−123.30–24.6)**	−64.3 to −11.5		−0.75		**0.0107**	
HB-REM		174.9 ± 198.3	**109.1 (3.00–715.20)**	72.0 ± 107.0	**29.5 (0.90–400.00)**	−102.9 ± 130.3	**−62.7 (−404.90–44.2)**	−178.2 to −27.6		−0.77		**0.0085**	
HB-NREM		127.6 ± 124.0	**95.8 (9.7–449.40)**	98.9 ± 94.5	**70.6 (5.50–353.40)**	−28.7 ± 37.7	**−13.4 (−96.90–20.90)**	−50.4 to −6.9		−0.77		**0.0085**	
Supine HB		187.3 ± 143.2	**157.4 (19.3–553.70)**	149.9 ± 128.7	**114.3 (11.80–481.80)**	−37.3 ± 77.1	**−71.90 (−135.90–121.40)**	−83.9 to 9.3		−0.45		0.1677	
Non-supine HB		112.5 ± 121.80	**73.4 (4.5–424.30)**	62.9 ± 94.4	**14.1 (0.00–352.80)**	−49.6 ± 74.8	**−8.9 (−246.60- −0.9)**	−92.8 to −6.4		−0.90		**0.0001**	

*HB* Hypoxic burden, *HB NREM* Hypoxic burden during NREM sleep. Comparison performed using the Wilcoxon test for paired samples; *HB REM* Hypoxic burden during REM sleep, *HB TST* Hypoxic burden during total sleep time, *AHI* Apnea-Hypopnea Index, *RDI* Respiratory Disturbance Index, *BMI* Body Mass Index, *T90* Percentage of total sleep time with oxyhemoglobin saturation < 90%, *95% CI* 95% confidence interval of the mean difference (Δ). Effect size: rank-biserial correlation. All HB values are expressed as %·min/h

¹Non-parametric distribution; ²p-value: statistical significance *p* < 0.05. Δ: Difference between postoperative and preoperative values (post − pre)

 In the analysis of postoperative HB-TST values among the 27 patients with complete paired assessment, 70.4% had HB-TST values below 53%·min/h, while 74.1% were below 73.1%·min/h and 77.8% were below 88%·min/h (Table 5). Among patients classified as surgical failures, 42.9% had postoperative HB-TST values below 53%·min/h and 57.1% below 88%·min/h (Table 5).

**Table 5:** Analysis of postoperative HB-TST in the overall group of patients with available preoperative and postoperative HB-TST values (N = 27) and subgroup with surgical failure (N = 14)

Postoperative HB-TST (overall group)	Patients (%)
<53 %·min/h	70.40
<73.1 %·min/h	74.10
<88 %·min/h	77.80
<97.1 %·min/h	77.80
Postoperative HB-TST (surgical failure)	
< 53 %·min/h	42.90
< 73.1 %·min/h	50.00
< 88 %·min/h	57.10
<97.1 %·min/h	57.10

*HB-TST * hypoxic burden during total sleep time

## Discussion

Among the main findings of this study, patients undergoing LP demonstrated significant reductions in HB across all variants, including HB-TST, HB-REM, HB-NREM, and both supine and non-supine positions. Furthermore, these reductions were also observed in patients classified as surgical failures according to Sher’s criteria, with reductions in HB-REM, HB-NREM, and non-supine HB, suggesting that therapeutic response to LP may not be fully captured by AHI alone.

The significant reduction in AHI and RDI after LP reinforces the effectiveness of the procedure. Although median postoperative AHI reached values compatible with surgical success, the overall success rate was lower than that reported in recent series [18, 21–23]. This may be related to selection bias resulting from the requirement for complete polysomnographic data suitable for HB calculation. Nevertheless, this finding highlights the relevance of the observed physiological improvements even in a population with lower surgical success rates.

Palatine tonsil grade was not significantly associated with surgical success, suggesting that the therapeutic response to LP may not depend exclusively on anatomical factors, but also on physiological and functional mechanisms involved in OSA pathophysiology.

The reduction in HB during REM sleep has important pathophysiological relevance, considering that REM sleep is characterized by greater muscular atonia and increased propensity for pharyngeal collapse [24, 25]. The significant reduction in HB-REM suggests that LP reduced the severity of desaturation events during the period of greatest upper airway vulnerability.

A significant reduction in HB was observed in both supine and non-supine positions. Since the supine position is associated with increased propensity for pharyngeal collapse and greater OSA severity, these findings suggest that LP promoted substantial upper airway stabilization regardless of body position [25]. Furthermore, the supine position represents one of the main polysomnographic phenotypes of OSA [26].

These findings are particularly relevant given the recognized limitations of AHI in stratifying OSA severity regarding cardiovascular outcomes and mortality [7]. Several recent cohort studies have demonstrated that HB more accurately captures exposure to nocturnal hypoxemia because it integrates the depth, duration, and frequency of obstructive respiratory events into a single metric [7, 8].

Recent studies have demonstrated associations between elevated HB and increased cardiovascular risk, heart failure, and mortality [11, 12, 27, 28]. In this context, the consistent HB reduction observed after LP suggests that surgical benefits may be more appropriately assessed using HB than AHI alone.

Although no consensus currently exists regarding ideal HB cutoff values for cardiovascular risk stratification, several cohorts have identified increased risk in patients with HB above 53%·min/h, 73.1%·min/h, and 88%·min/h [8, 9, 12, 29]. When postoperative HB-TST values were evaluated according to these cutoff points, 70.4%, 74.1%, and 77.8% of patients achieved values below the respective thresholds. These findings illustrate that even in a population with 55.3% surgical success according to Sher’s criteria, many patients reached HB ranges previously associated with lower cardiovascular risk. However, cardiovascular outcomes were not assessed in the present study.

As expected, patients classified as surgical successes demonstrated greater reductions in some respiratory variables compared with the surgical failure group. However, the absence of relevant differences in certain HB variables raised the hypothesis that patients classified as surgical failures might also achieve clinically relevant pathophysiological improvement.

The isolated analysis of the surgical failure group demonstrated a significant reduction in HB-TST, HB-REM, HB-NREM, and non-supine HB, despite the absence of a statistically significant reduction in AHI. Furthermore, a substantial proportion of these patients presented postoperative HB values below cutoff points previously associated with increased cardiovascular risk, with 42.9%, 50.0%, 57.1%, and 57.1% of patients below these thresholds. These findings should be interpreted as changes in a physiological marker associated with cardiovascular outcomes.

Considering that Sher’s criteria use exclusively AHI to define surgical success, these findings suggest that some patients classified as surgical failures may still demonstrate clinically relevant pathophysiological improvement.

These findings reinforce that therapeutic response in OSA may not be fully captured solely by respiratory event frequency. Alternative metrics such as HB may more adequately represent the pathophysiological burden of the disease and potentially contribute to future definitions of surgical success [7, 28, 30].

The present study has limitations. First, the reduction from the initially identified cohort of 118 patients to the final paired HB analysis of 27 patients may have introduced selection bias. Because the paired HB analysis was restricted to patients with complete preoperative and postoperative data available for HB calculation, the final cohort may have differed from patients who were not included due to incomplete records, unavailable follow-up PSG, or software incompatibility with Sleepware preventing HB calculation. Consequently, the findings may not fully represent the overall population undergoing lateral pharyngoplasty, and their generalizability should be interpreted with caution. In addition, no prior sample size calculation was performed due to the unprecedented nature of the study. Furthermore, the mixed retrospective and prospective design limits adequate control of confounding variables and increases the possibility of information bias.

Despite these limitations, to date, no studies have evaluated HB behavior after LP. In addition, the paired preoperative and postoperative analysis allowed intraindividual assessment of therapeutic response, thereby reducing interindividual variability. The use of polysomnographic studies performed in the same laboratory, interpreted by the same specialist, and combined with a standardized surgical technique contributed to greater methodological homogeneity. Despite the relatively small sample size for paired HB analysis, large effect sizes were observed for HB reduction both in the overall group (rank-biserial correlation = − 0.92) and in the surgical failure subgroup (rank-biserial correlation = − 0.75). These findings reinforce the pathophysiological relevance of the observed changes and suggest clinically meaningful improvement even among patients who did not meet Sher’s criteria based exclusively on AHI.

## Conclusion

Patients with OSA undergoing lateral pharyngoplasty demonstrated consistent and significant reductions in hypoxic burden during total sleep time, REM sleep, NREM sleep, and both supine and non-supine positions. Patients classified as surgical failures according to Sher’s criteria also demonstrated statistically significant reductions in HB-TST, HB-REM, HB-NREM, and non-supine HB. HB reduction was more pronounced in patients classified as surgical successes according to Sher’s criteria.

These findings support the hypothesis that HB represents a relevant complementary marker for evaluating surgical therapeutic response, with potential application in cardiovascular risk stratification in patients with OSA. Prospective longitudinal studies are warranted to determine whether the reduction in hypoxic burden following lateral pharyngoplasty translates into improvements in cardiovascular outcomes.

## Data Availability

The datasets generated during and/or analyzed during the current study are available from the corresponding author on reasonable request.
